# Beneficial osseointegration effect of hydroxyapatite coating on cranial implant – FEM investigation

**DOI:** 10.1371/journal.pone.0254837

**Published:** 2021-07-19

**Authors:** Jakub Chamrad, Petr Marcián, Jan Cizek

**Affiliations:** 1 Department of Solid Mechanics, Mechatronics and Biomechanics, Brno University of Technology, Brno, Czech Republic; 2 Institute of Plasma Physics of the Czech Academy of Sciences, Prague, Czech Republic; University of Vigo, SPAIN

## Abstract

A firm connection of the bone-implant-fixation system is of utmost importance for patients with cranial defects. In order to improve the connection reliability, the current research focuses on finding the optimal fixation method, as well as selection of the implant manufacturing methods and the used materials. For the latter, implementation of bioactive materials such as hydroxyapatite or other calcium phosphates has also been considered in the literature. The aim of this study was to investigate the effect of gradual osseointegration on the biomechanical performance of cranial Ti6Al4V implants with a deposited HA coating as the osseointegration agent. This effect was assessed by two different computational approaches using finite element method (FEM) modeling. The values of key input parameters necessary for FEM were obtained from experimental plasma spray deposition of HA layers onto Ti6Al4V samples. Immediately upon implantation, the HA layer at the bone-implant contact area brought only a slight decrease in the values of von Mises stress in the implant and the micro-screws when compared to a non-coated counterpart; importantly, this was without any negative trade-off in other important characteristics. The major benefit of the HA coatings was manifested upon the modeled osseointegration: the results of both approaches confirmed a significant reduction of investigated parameters such as the total implant displacements (reduced from 0.050 mm to 0.012 mm and 0.002 mm while using *Approach I* and *II*, respectively) and stresses (reduced from 52 MPa to 10 MPa and 1 MPa) in the implanted components in comparison to non-coated variant. This is a very promising result for potential use of thermally sprayed HA coatings for cranial implants.

## 1. Introduction

Cranioplasty (reconstruction of defects of a skull) is a surgical procedure that, apart from restoration of the patient’s appearance, repairs bone defects, thereby contributing to mechanical protection of the most important organ in the human body, the brain [1]. Such procedure involves integration of an implant and its fixation using mini-plates and micro-screws (screws with micro-threads) to prevent the implant displacements. Naturally, selection of a suitable implant and its fixation is essential for the successful performance [2, 3]. A significant progress in this area was enabled by implementation of so-called patient-specific *(PS)* approach. Such approach allows to individually design the implants to obtain a customized fit for each patient, which results in a greater accuracy, shorter rehabilitation and versatile pre-operative planning [4].

Recently, the PS approach in the field of implantology (including cranioplasty [5]) was facilitated by implementation of additive manufacturing *(AM)* technologies. The most frequently used 3D-printable materials for implants in cranioplasty are poly-methyl methacrylate *(PMMA)* [2, 5–8]; polyether ether ketone *(PEEK)* [5, 7, 9]; and titanium alloys (such as Ti6Al4V, Ti6Al7Nb) [1, 7, 9].

Due to the biocompatibility and the strength requirements, the fixation components are usually manufactured of titanium alloys only [1, 5, 10, 11]. Nowadays, two different concepts of fixation are available: fixation mini-plates, and overlapping margins with pre-drilled holes [1]. The former is a versatile solution, bridging the implant and the skull bone, while the latter should result into a stronger connection to the bone. Despite the progress in the methods and materials, the fixation system of the implant is the weakest element [12] and efforts toward its improvement need to be realized. One of the potential ways to further improve the fixation between the implant and the bone is to stimulate osseointegration by migration of osteogenic cells to the bone defect [13]. Recently, the positive effect of osseointegration led scientists to study cranial implants partly made from bioactive materials [14, 15]. Same authors in the study [7] observed bone formation at bone-implant contact (BIC) interface using bioactive ceramics as a component of cranial implants.

Osseointegration stimulation can be also realized by an additional surface treatment, such as deposition of bioactive coatings. Such approach combines the strength, ductility and availability of the core metallic (often titanium alloy) implants with enhanced osseointegration between the bone and the porous implant coating material due to its ability to bond directly to the bone [16].

In orthopedics, hydroxyapatite *(HA)* coatings have been successfully used to improve the implant fixation [17, 18]. Among other, the porous HA coatings have been proven to have a positive impact on the healing time [19] and the strength of the implant fixation [20, 21]. Traditionally, these porous coatings are produced by thermal spray deposition technologies, in particular by the plasma spray method [22, 23]. This method appears to be beneficial from the point of bio-corrosion resistance, chemical control [24] and process efficiency [22]. On the other hand, the method has disadvantages stemming mostly from the elevated processing temperatures such as microstructural heterogeneity and lower HA crystallinity [25–28]. Also, the plasma spray processing temperatures inherently disallow using polymer-based materials. That said, titanium alloys present an appropriate choice as HA-coated implant for cranioplasty, for they are capable to withstand the high processing temperatures and are also relatively easy to be 3D-printed in order to meet the PS approach requirements.

The aim of this study was to assess and investigate the impact of added HA coatings on monitored biomechanical parameters of the Ti6Al4V cranial implant with its fixation components using FEM. The input data for the FEM calculations (such as, e. g., the value of Young’s modulus) were partially obtained from realized experiments of plasma spraying of HA coatings onto Ti6Al4V samples and subsequent analyses. This approach of auxiliary fixation of the implanted device to the bone by means of applied HA coating is well-known e.g. in the orthopedics field of hard tissue implants for joints. However, it is not commonly used in relation to cranial implantology, either in a clinical practice or in the scientific literature. This paper presents an initial attempt to study the potential benefits of implementing the procedure in the field of cranioplasty.

## 2. Materials and methods

Experimental spray deposition of HA coatings onto Ti6Al4V substrates was realized in order to gain relevant data about the coatings. This involved optimization of the deposition conditions (e.g., the stand-off distance) by observation of deformation of individual HA particles upon their impact. Subsequently, the coatings deposited under the optimized conditions were mechanically tested, providing the actual material properties to the subsequent FEM modeling. In the modeling, two different approaches were introduced to assess osseointegration process of cranial implants with HA coating at bone-implant contact (*BIC*).

### 2.1. Deposition of HA coatings, evaluation of properties

To aid the FEM modeling, an experimental study was carried out. In the process, HA coatings were plasma spray deposited onto commercial purity Ti6Al4V Gr 5 alloy substrates (Bibus Metals, Czech Republic). Twenty plates of dimensions 20 × 20 mm^2^ and thickness 5 mm and six plates of dimensions 20 × 60 mm^2^ of thickness 5 mm were used. The substrates were grit blasted using Al_2_O_3_ particles to an average surface roughness of *R*_*a*_ = 5.27 μm and then cleaned in acetone using ultrasonic bath for at least 5 minutes. For the selection of an appropriate stand-off distance experiments, 20 × 60 mm^2^ borosilicate glass substrates were used in a non-grit blasted state.

A spray-dried HA powder of average particle sizes of 61–158 μm and > 98% purity was obtained from MediCoat company (MediCoat AG, Switzerland). According to the manufacturer, the powder exhibited > 95% crystallinity and Ca/P ratio 1.66–1.72. Owing to its precipitation production method, the atomized powder agglomerates exhibited spherical morphology.

The deposition was carried out using a unique hybrid water-stabilized plasma torch (WSP-H500) available at Institute of Plasma Physics of the Czech Academy of Science. As opposed to the traditional gas-stabilized torches, the plasma in WSP-H500 is formed from deionized water tangentially rotating along the inner walls of the torch chamber. The arc is stabilized between a tungsten cathode and a revolving copper anode located in front of the torch nozzle orifice. More details on the technology can be found e.g. in [29].

The torch was operated at 500 A current, corresponding to approximately 150 kW of net power. The argon flow was kept at 15 slpm. Based on previous experience with the spraying of HA, the feed distance (i.e., distance from the plasma jet beginning and the area of material insertion into the jet) was selected as 60 mm. The torch was mounted onto a robotic arm and the spraying was realized using the transversal speed of 30 mm/s. In all runs with Ti6Al4V substrates, the temperature was monitored using thermo-camera and also a thermocouple attached to the rear side of one of the samples. Using this monitoring, the substrates were initially pre-heated to 220°C (prior to the first deposition). Using a mounted set of air-blades, they were then cooled to 120°C between all remaining individual torch passes. This was done to prevent excessive heating of the metal that could potentially jeopardize a number of properties (such as e.g. phase composition, adhesion).

**Fig 1:**
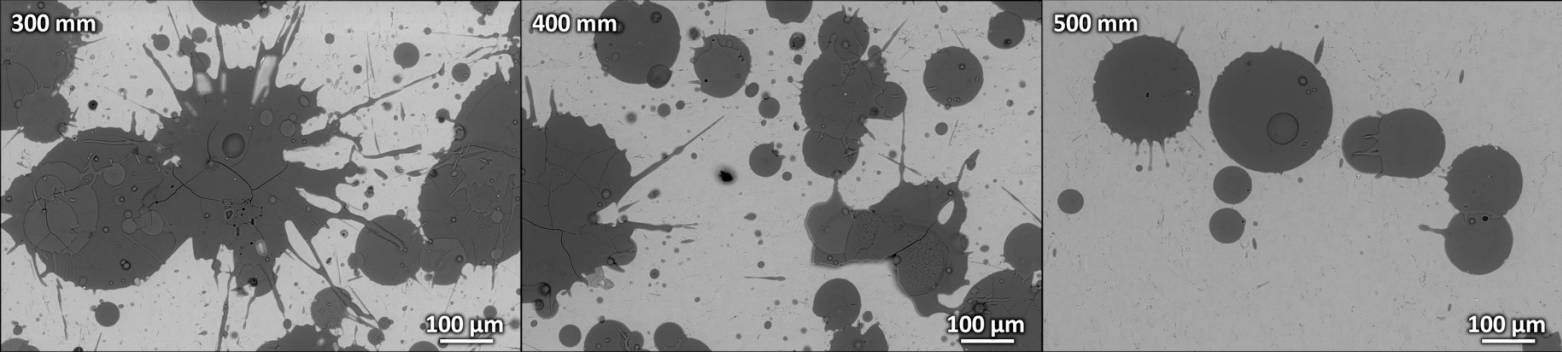
Selection of an appropriate stand-off distance during the HA coatings deposition process: Single splats spreading behavior upon plasma spraying at different stand-off distances 300 mm, 400 mm and 500 mm. Pictures obtained using scanning electron microscope (back-scattered electrons).

To determine the appropriate stand-off distance for spraying of these coatings (distance between the torch and the substrates), a complementary test was done first: the glass substrates were positioned at different stand-off distances (300, 400 and 500 mm) and, using rapid, single-pass movement of the robotic arm with the WSP-H torch, single splats (Fig 1) were collected to study their spreading behavior and potential cracking (reliable indicators of the future coating quality). To meet the conditions used for the subsequent coatings deposition, the substrates for this preliminary testing were, as well, preheated prior to the splats collection. The results have shown that the splats deposited at shorter stand-off distances (300 and 400 mm) were too hot, which translated into their splashing and cracking upon impact and solidification (Fig 1). At the distance of 500 mm, non-splashed splats formed, yielding a favorable round shape with uninterrupted rims and no porosity in the center of the splats. Based on the results, the stand-off distance of 500 mm was used for all subsequent coatings deposition.

Two HA coatings sets were then deposited using 8 and 16 passes of the plasma torch, resulting into two different thicknesses of the sets (approximately 160 μm and 320 μm).

Nanoindentation measurements were subsequently performed to obtain Young’s modulus of the deposited HA coatings. Fifteen measurements were done under the load of 100 mN. The force was selected with respect to the material porosity and its highly heterogeneous structure. The surface for nanoindentation was precisely polished into the roughness of *R*_*a*_ < 1 μm.

### 2.2. Computational modeling

#### 2.2.1. Geometry model

**Fig 2:**
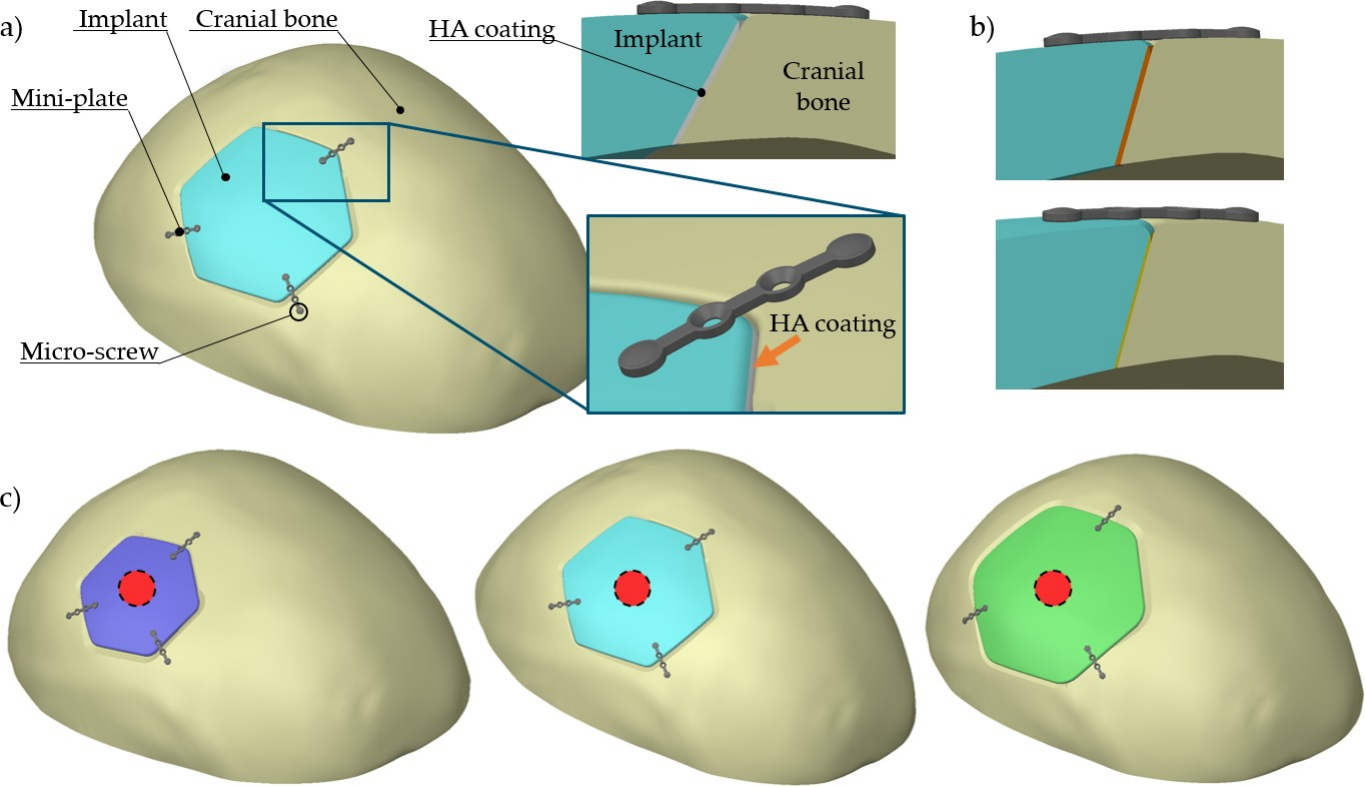
a) Cranium with the modeled medium-size defect. The magnified inset shows the Ti6Al4V implant detail including the HA coating deposited onto its rim, and the fixation mini-plate and micro-screws. (b) Two different thicknesses of HA coating (320 μm–orange and 160 μm–yellow). (c) Three different sizes of the cranial implant, small, medium, and large. The circular red areas (~ 310 mm^2^) correspond to the location of the applied external force caused by the head weight (50 N).

In this study, a Computed tomography (*CT)* data of an undamaged male skull in the physiological state was obtained from the male datasets of the Visible Human Project [30]. The voxel size was 0.33 × 0.33 × 1.0 mm^3^. The CT data were imported into *STL Model Creator* (Matlab 2012, Math Works, USA) image processing software developed by the authors [31, 32] to obtain 3D standard tessellation language *(STL)* file using both automatic and manual segmentation. The obtained 3D STL file therefore contained information about the external geometry of the cranium. The STL file was then processed in 3D CAD software *Catia* (CATIA V5, 2016, Dassault Systèmes, France), where the volume model was created. Three artificial defects differing in their relative size (small, medium and large; Fig 2C) were created in *SolidWorks* (SolidWorks 2012, Dassault Systèmes, France) on the right side of the cranium, in a location that does not cross the sagittal plane. The aim of this study was not to assess specific implant shapes for a clinical study; therefore, the shape of the implant was chosen so that it did not have a circular shape, but contained edges as potential risk locations instead. Correspondingly, three different implant sizes (~ 12.5 cm^3^, ~ 18.8 cm^3^ and ~ 27.4 cm^3^) were designed (Fig 2). The medium defect was modeled first, with the other two sizes created using the equidistant tool. Following that, three fixation mini-plates and six screws with micro-thread were modeled to prevent the implant movement.

Given the total of six combinations (three implant sizes and two HA coating thicknesses), the individual computed combinations were denoted as *X*_*YY*Y_, where *X* corresponds to size of the implant (S = small, M = medium, L = large) and the subscript *YYY* describes the coating thickness in microns (160, 320).

The added HA coating was modelled at the bone–implant contact *(BIC)* located on the implant’s side. That said, the coating did not cover the entire implant surface. Instead, the HA was modeled along the peripheral surface of the implant only. In analogy with the performed experimental work, two coating thicknesses were modeled, 160 and 320 μm (Fig 2B).

#### 2.2.2. FE mesh

The 3D geometry of the individual components was assembled and discretized in Ansys (*ANSYS Academic Research Mechanical*, *Release 19*.*0*, Swanson Analysis Inc., USA). All volumes were discretized using a quadratic hexahedral, 10-node element type (SOLID187). All contacting parts were connected using 3D contact elements TARGE160 and CONTA174 (frictional contact) with a conservative friction coefficient (*FC*) of 0.05. The connection between the HA coating and the implant was assumed as bonded, in accordance with the superior adherence of the real plasma sprayed HA coatings [33].

**Fig 3:**
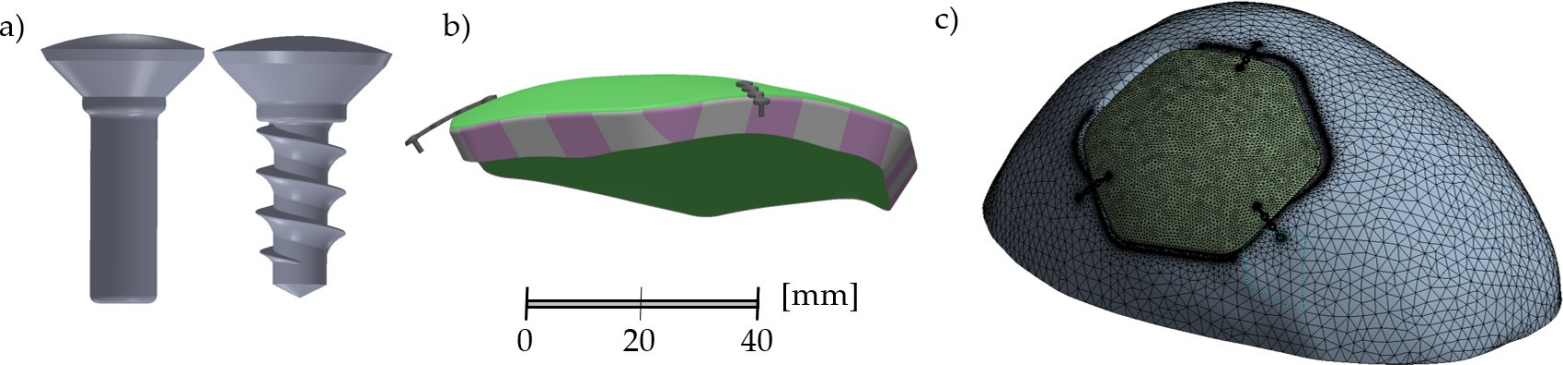
(a) The cylindrical thread-less and screws with micro-thread used in the coarse and sub-model computations, respectively. (b) Illustration of the sub-areas division of the peripheral HA coatings (pink-grey) used to simulate the implant (green) osseointegration in *Approach II*. (c) FE mesh of coarse model.

A two-stage modelling strategy was applied to shorten the overall computing time [8]:

- **Coarse model**: this model was used as a first approximation and modeled the whole cranium. Simplified, cylindrical thread-less screws were connected to the cranial bone or the implant using a bonded contact [8], as shown in Fig 3A. The total amount of elements was approximately 1.56M, corresponding to 2.63M nodes (FE mesh is shown in Fig 3C).
- **Sub-model**: Subsequently, a more detailed modeling was performed for critical parts obtained from the coarse model. Screws with micro-thread (cf. the thread-less screws in the coarse step) were used for the connection with the implant or the cranial bone. Frictional contact with a conservative *FC* of 0.05 was assumed. The total number of the elements was 1.44M, which corresponded to 2.17M nodes.

The BIC (contact between the bone surface and the HA surface, in this case) was modeled as frictional at first. The osseointegration effect brought by the introduction of the HA coating was then modeled using two different approaches:

- **Approach I:** A study [34] models osseointegration by increasing a value of the friction factor. The factor is labeled as Friction Coefficient (*FC)* in Ansys. In this approach, the osseointegration was modeled via series of computations using gradual increase of the FC at BIC. The increase ranged from the frictionless minimum (*FC* = 0.05, corresponding to non-integrated implant) to *FC* = 2.00, and further to a rough contact (which is defined as contact with an infinitely high value of FC in Ansys WB software) was analyzed as well.
- **Approach II:** The peripheral HA coating was divided into pre-defined smaller sub-areas (Fig 3B) to mimic the gradual bone ingrowth and its mineralization [35]. In this approach, the first computation identified the sub-area with the lowest relative displacement. Subsequently, the character of the contact at BIC interface for this sub-area was changed from the frictional to bonded. The calculation was then performed again and a new sub-area with the lowest displacements was identified. The procedure was repeated until all sub-areas were bonded at BIC interface.

#### 2.2.3. Material model

All materials were modelled as linear, homogeneous, isotropic and elastic. This assumption presents a simplification as compared to the real materials. However, it is a rather standard practice for modeling the implants [2, 12, 36]. The HA thin layer has not been commonly modeled in cranial implantology, so samples with this layer were sprayed and analyzed to obtain relevant input values. All material properties are listed in Table 1.

**Table 1:** Mechanical properties of the used materials.

	Young’s Modulus [GPa]	Poisson’s Ratio [–]	Yield stress [MPa]	References
Cranial bone	4.1	0.21	122	[37–39]
Ti6Al4V	110.0	0.30	847	[40]
HA	71.8^1^	0.30	50	[41, 42]

^1^The Young’s modulus was obtained from nanoindentation testing of real coatings (see section 3.1).

#### 2.2.4. Loads and boundary conditions

Several studies showed that, as opposed to unloaded cases, a functional loading may enhance osseointegration by expanding the direct BIC areas [35, 43]. Therefore, two loads were assumed to act in this study: intracranial pressure *(ICP)* and static force. The modeled value of ICP was 2 kPa (15 mmHg), which corresponds to the physiological value [44]. The ICP acts on the inner side of the cranial bone and the implant. The external static force was modeled as 50 N, corresponding approximately to the weight of a head [8]. The force was applied to a small circular area in the center part of the implant (denoted as red regions in Fig 2C). All analyzed models were fixed at the bottom side of the modelled part of the cranium [36].

## 3. Results

### 3.1. Deposited HA coatings

The total coating thickness measured from the scanning electron microscope (SEM) cross-section images after 8 or 16 plasma torch passes reached 160 μm and 320 μm, respectively. This corresponded to a steady, linear increase in thickness of approximately 20 μm per torch pass.

The Ca/P ratio in the coatings determined via energy-dispersive X-ray spectroscopy (EDX) reached 2.45. This is markedly higher than that of the purchased HA powder (1.89) and the theoretical value of 1.69.

**Fig 4:**
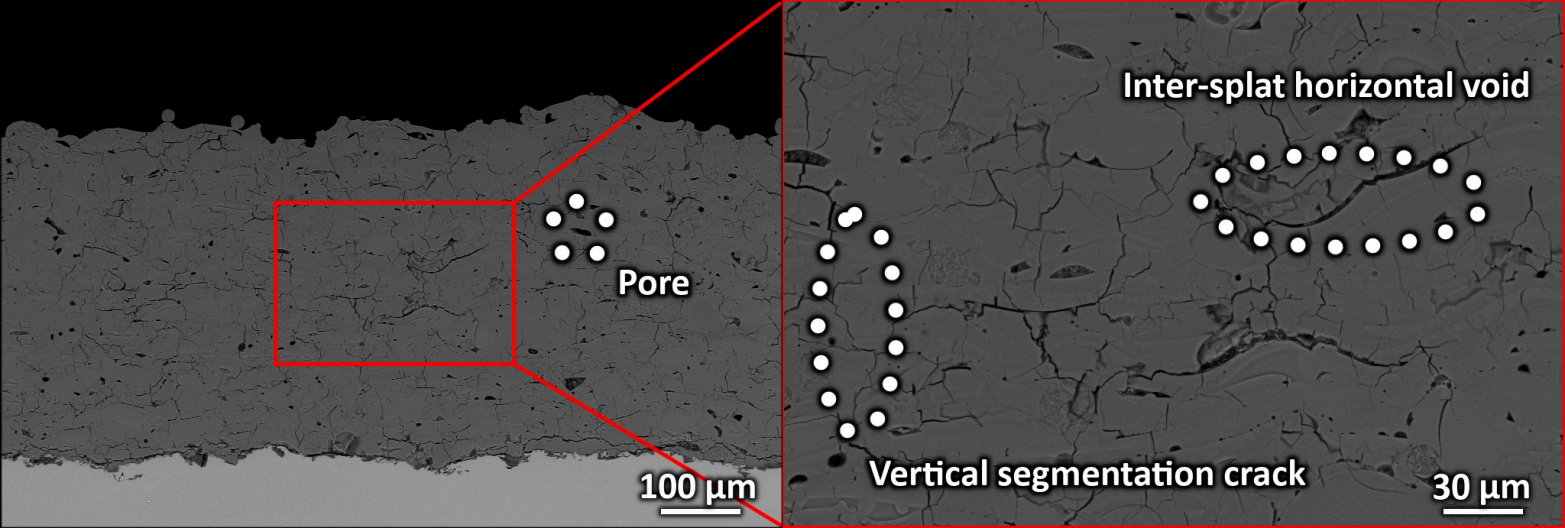
Microstructure of the HA coatings produced on Ti6Al4V substrate (sample T_320_ shown). Given the thermal spray production method, the coating contained some pores, voids and cracks. The magnified inset shows network of inter-splat voids forming by improper droplet spreading and contact, as well as vertical segmentation cracks in the coating, a consequence of internal stresses alleviation upon cooling to room temperature.

The coatings exhibited dense microstructure consisting of well-connected splats (Fig 4), with only infrequent horizontal inter-splat voids. In fact, the individual splat boundaries were difficult to distinguish in the structure, indicating good mechanical properties of the coatings. This was partially a consequence of the initial process of selection of an appropriate stand-off distance. A network of vertical segmentation cracks can be seen throughout the coating (inset of Fig 4), originating from alleviation of internal stresses upon cooling of the substrate-coating system to room temperature. The cracks were trans-splat and did not follow the splat boundaries, indicating a good coating quality again. The average porosity of the coatings was determined by two methods. The SEM image analysis indicated an internal porosity value of 8.0 ± 1.0%. The open porosity measured via Archimedean weighing method reached 4.6%.

Nanoindentation procedure was employed to accurately measure the Young’s modulus of the HA coatings. The average value reached 71.8 ± 6.4 GPa. The loading and unloading curves from the measurement are shown in *Appendix A* in S1 Appendix (eight valid indentations).

### 3.2. Computational modeling

In this study, the maximum values of total displacement of the cranial implants (coarse model) and von Mises stresses of implants, fixation mini-plates, and micro-screws (sub-model) were investigated. The computations were performed for different level of osseointegration. The non-osseointegrated state was titled as an *as-fixed state* of the coated implant. The other investigated states of the coated implant were partly or fully osseointegrated in both applied approaches.

#### 3.2.1. Approach I: Changing the frictional coefficient at BIC

**Fig 5:**
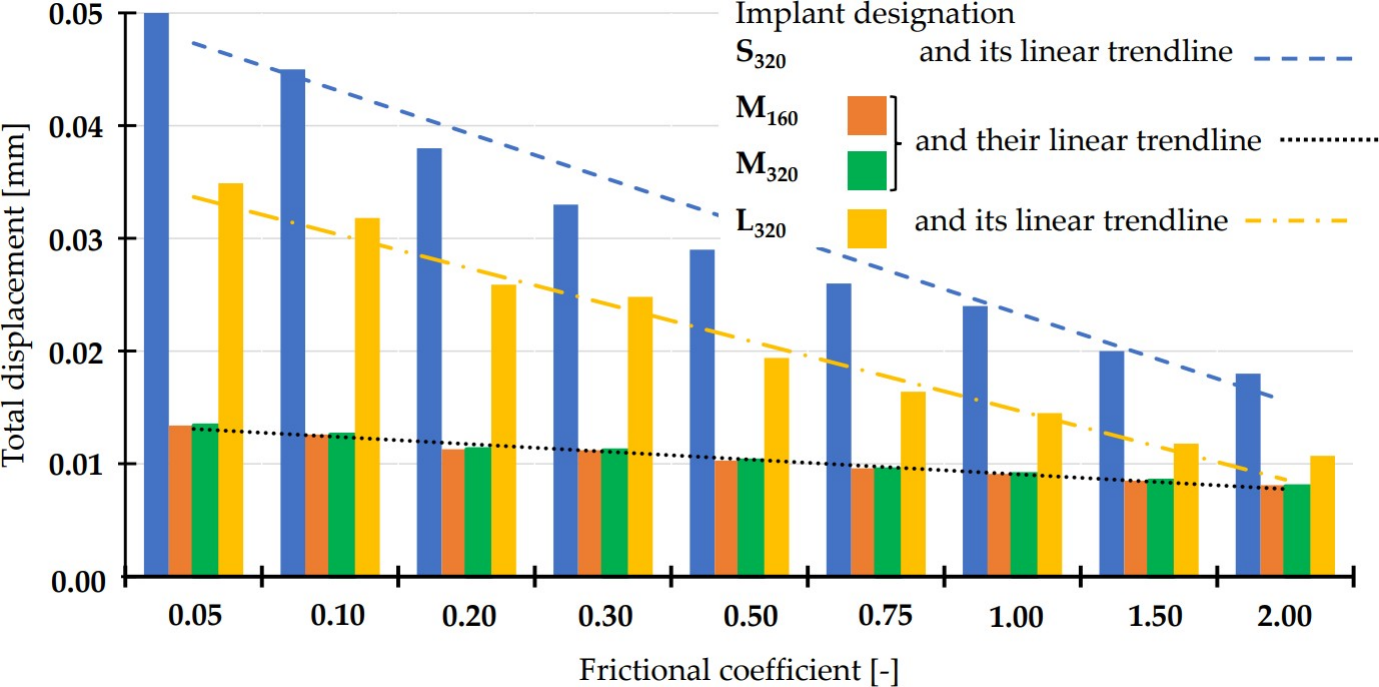
Reduction in the total implant displacements for different implant sizes with increasing frictional coefficient at BIC surface area computed according to *Approach I*. The increase in frictional coefficient simulated osseointegration in *Approach I*.

*3*.*2*.*1*.*1*. *Total displacement of the implant*. The maximum values of the total displacement of the implant (i.e., regions of the implant where the displacement recorded the maximum values for a given combination of *FC* value and implant size) are demonstrated in Fig 5.

**Fig 6:**
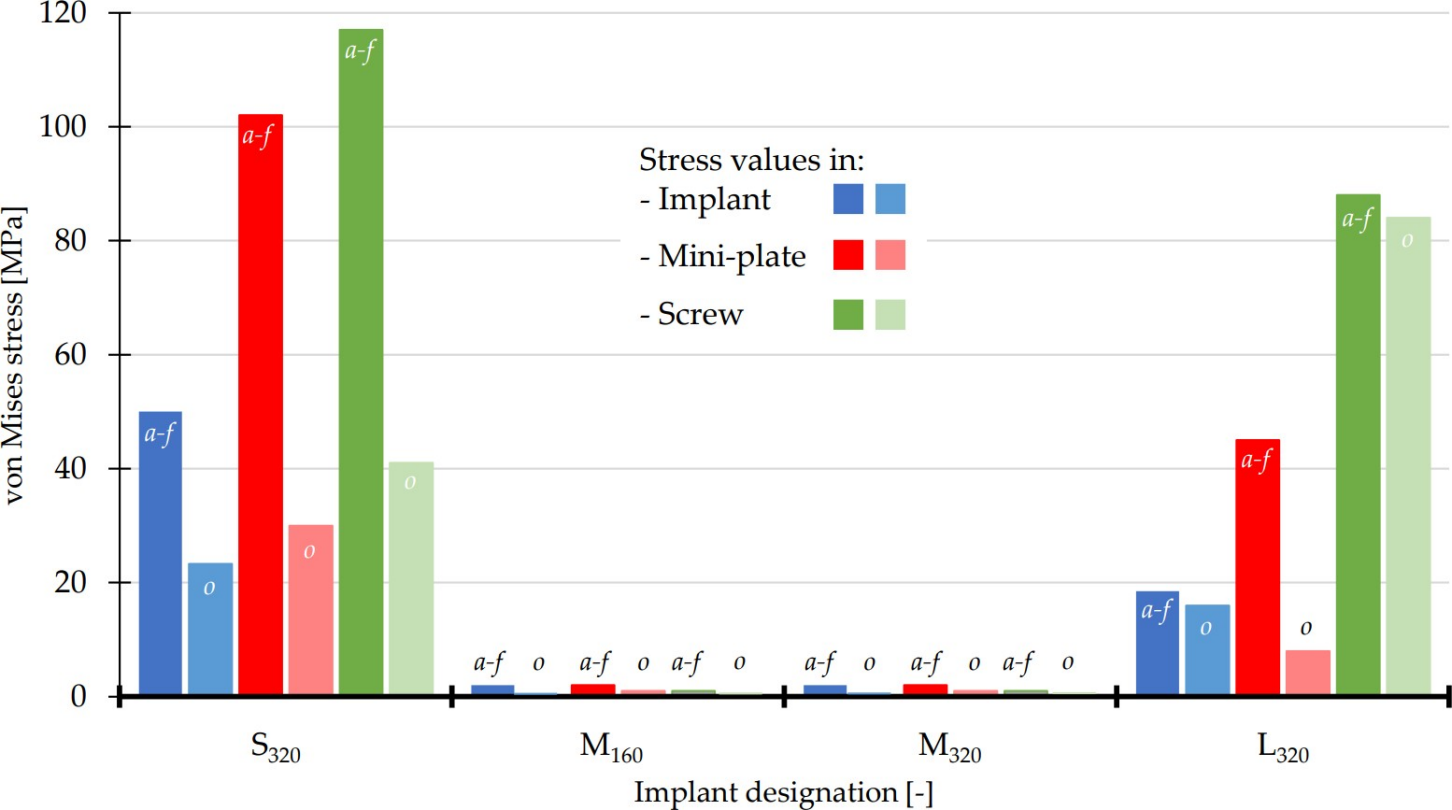
The maximum values of von Mises stresses in implant (blue colors), fixation mini-plates (red colors) and screws with micro-thread (green colors). The values of as-fixed state were labeled with ,,*a-f*” and the values of partly osseointegrated state (*FC* = 0.75) with, ,,o”.

The results showed that, before osseointegration, the small-size implants (represented by S_320_ in Fig 5) exhibited higher values of total displacement as compared to large and medium implants (0.050 mm of S_320_ vs. 0.035 of L_320_ and 0.013 mm for M_160_ and M_320_). With increasing of the *FC* to 0.75, all displacement values decreased to 0.026, 0.016, 0.001 and 0.001 mm for S_320_, L_320_, M_160_ and M_320_ implants. Upon such level of osseointegration, there were no big differences between the variants of thin (160 μm) and thick (320 μm) variants (therefore, only S_320_ and L_320_ variants are shown in Figs 5 and 6 to retain clarity; implant variants S_160_ and L_160_ behaved similarly to their 320 variants).

*3*.*2*.*1*.*2*. *Von Mises stress*. The values of von Mises stresses were computed for the entire fixation system, i.e., the implant, the fixation mini-plates, and the screws.

In the as-fixed (i.e. non-osseointegrated) state (*FC* = 0.05), the highest values of von Mises stress in the Ti6Al4V implant were observed in S_320_ (50 MPa; Fig 6). In all calculations, the von Mises stress in the implants reached the highest values in the threaded holes for the fixation screws (Fig 9). The lowest stress value was recorded for the medium-size implants M_160_ and M_320_, approximately 2 MPa. Upon the increase of *FC* to 0.75, the values of stresses decreased markedly in all implant variants (Fig 6, *blue bars*). The values of von Mises stress monitored in the HA coating were significantly below the material yield strength. In fact, the highest detected values in S_320_ and M_320_ were 1.5 MPa and 2.5 MPa, for the monitored *FC* = 0.05 and *FC* = 0.75, respectively.

The highest values of von Mises stress in the titanium fixation mini-plates were again observed in small implant (102 MPa). The lowest stress values were also obtained for M_160_ and M_320_ (2 MPa in both variants). Upon the increase of *FC* to 0.75, the values of stresses decreased markedly in all analyzed variants (Fig 6, *red bars*).

In accordance with the implants and fixation mini-plates, the highest values of stress in the micro-screws were observed in S_320_ implant (117 MPa). The lowest value was, yet again, observed in M–variants (1 MPa). Upon the increase of *FC* to 0.75, the lowest decrease was observed in L_320_ implant (from initial 88 MPa to 84 MPa).

#### 3.2.2 Approach II: Gradual osseointegration

**Fig 7:**
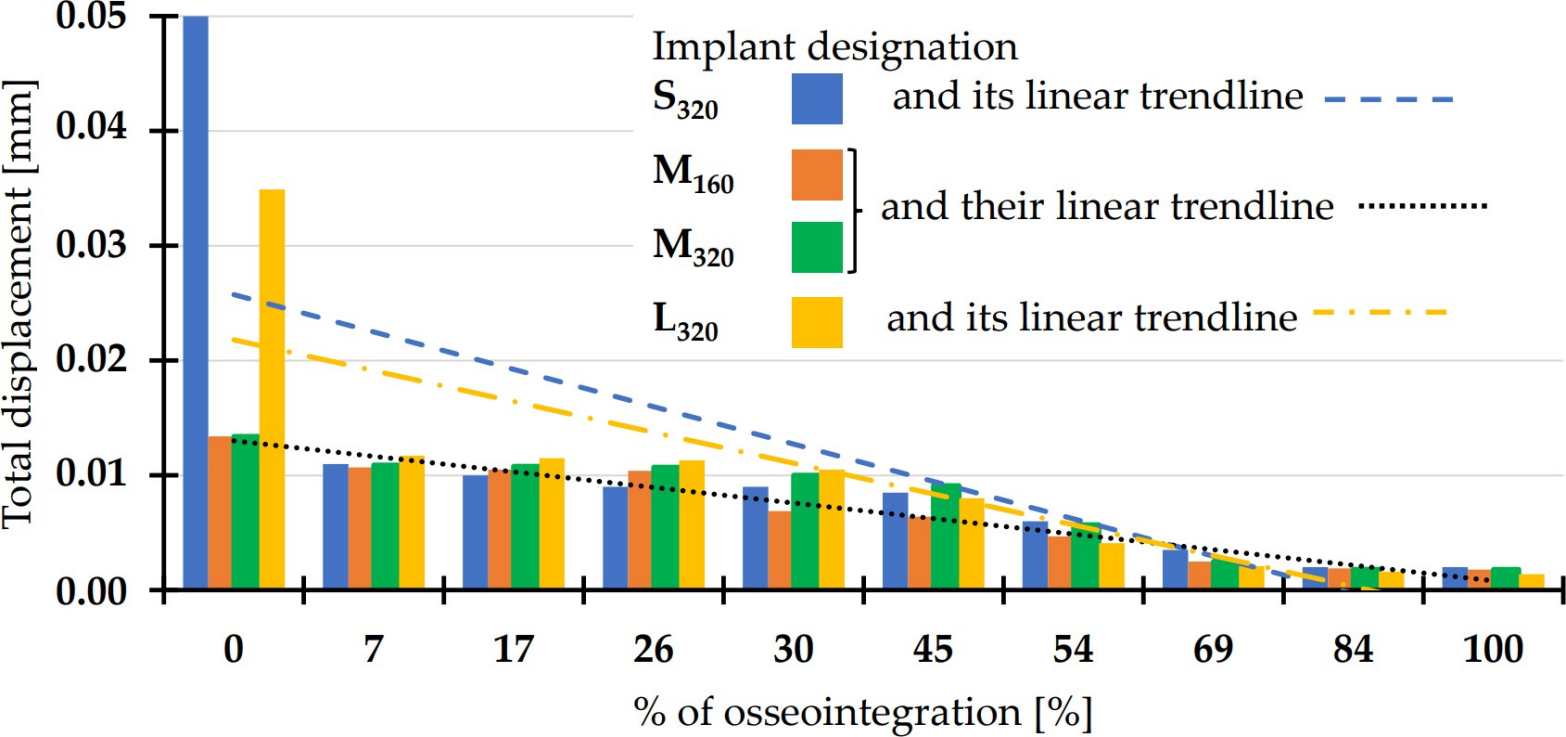
Reduction in the total implant displacements for different implant sizes with increasing osseointegration levels simulated in *Approach II*. The “% of osseointegration” was modeled by the percentage of the BIC surface area that was bonded.

*3*.*2*.*2*.*1*. *Total displacement of the implant*. The results of the as-fixed (i.e. non-osseointegrated) state were virtually identical to those obtained via *Approach I*. At approximately 30% osseointegration level (corresponding to 30% bonded contact and 70% frictional contact (*FC* = 0.05) at the BIC interface), the values decreased below 0.011 mm for all implant variants. Even in this approach, the differences between the thin (160 μm) and thick (320 μm) variants were negligible (Fig 7).

*3*.*2*.*2*.*2*. *Von Mises stress*. The computed von Mises stresses of the implant, the fixation mini-plates and the screws prior to osseointegration (0%) were similar to those obtained in *Approach I*.

**Fig 8:**
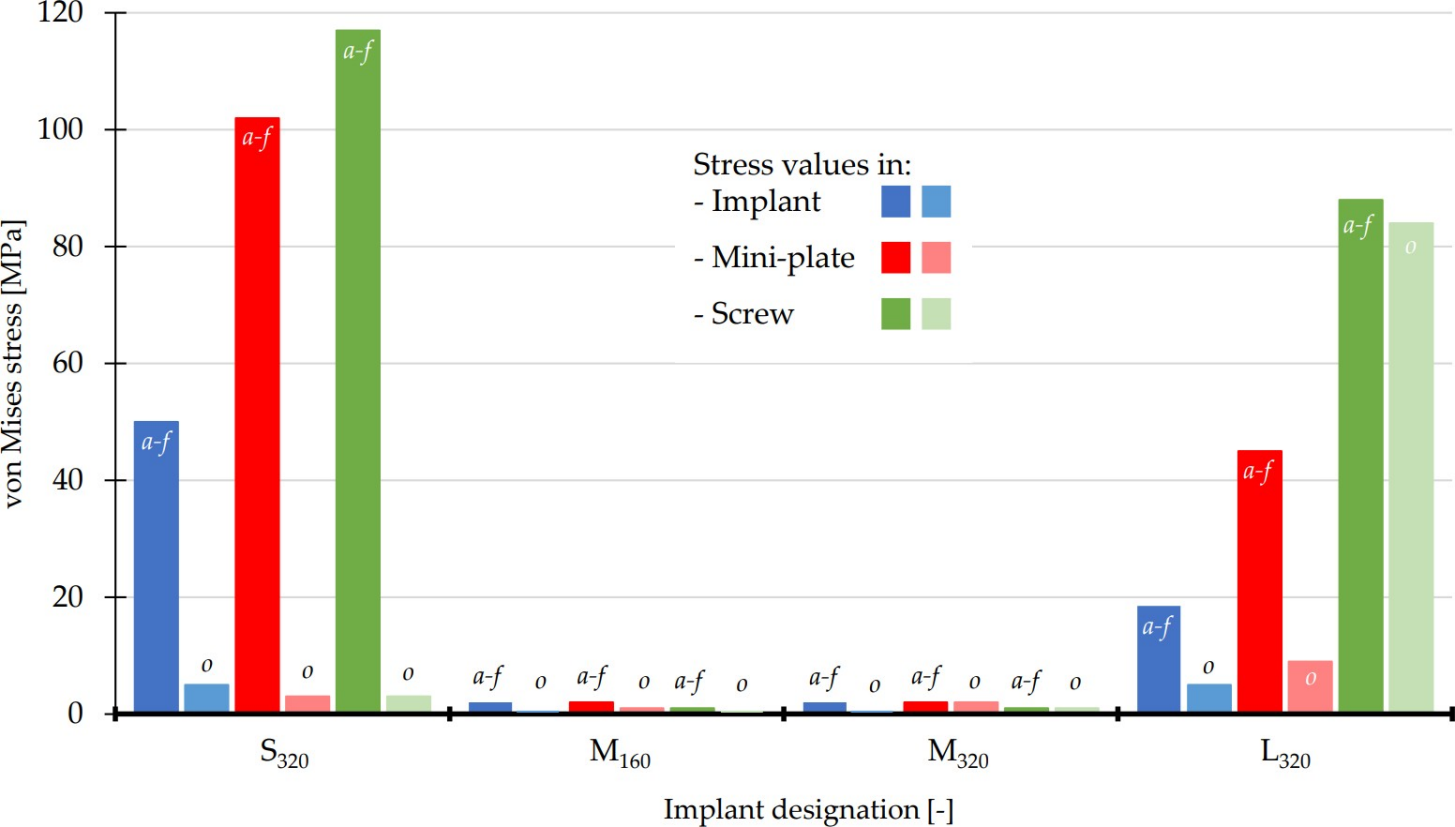
The maximum values of von Mises stresses in implant (blue colors), fixation mini-plates (red colors) and screws with micro-thread (green colors). The values of as-fixed state were labeled with ,,*a-f*” and the values of 30% osseointegration levels (30% of bonded contact at BIC) with ,,o”.

**Fig 9:**
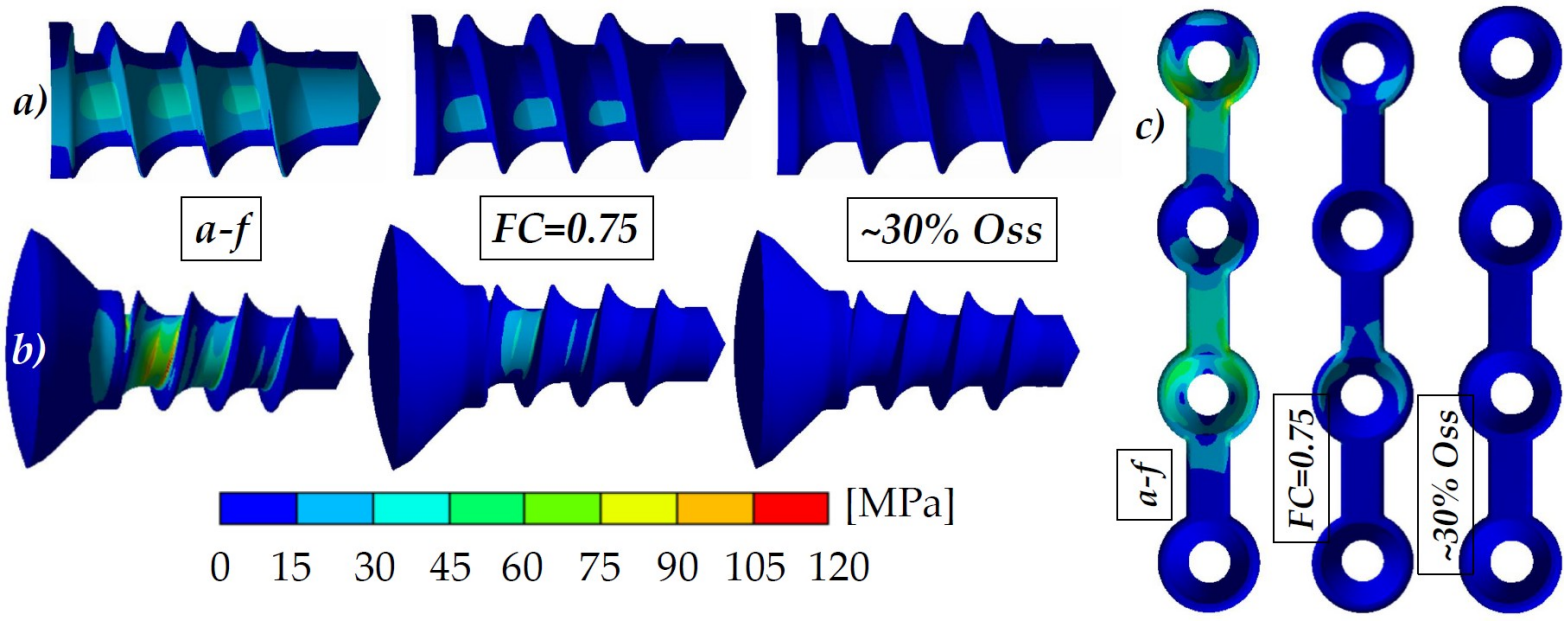
Typical results of von Mises stress distribution in (a) implant (implant holes) and fixation components (b—screws and c–mini-plates) for S_320_ variant. As-fixed states were labeled with ,,*a-f*”, the values of partly osseointegrated states were labeled with ,,*FC = 0*.*75”* (Approach I) and ,,*~30% Oss”* (Approach II).

In case of 30% osseointegration levels, the stress values in the Ti6Al4V implants significantly decreased in almost all variants; the highest values of von Mises stress in were observed in S_320_ and L_320_ implants (5 MPa), while the lowest values were recorded for M-size implants (see Figs 8 and 9). The values of von Mises stress in the HA coating in Approach II were also well below the material yield strength. The highest value was detected in L_320_ (5 MPa) for the 30% of osseointegration level.

Upon 30% of osseointegration, the highest value of von Mises stress recorded in the fixation mini-plates was detected for the L_320_ variant (9 MPa), while the lowest values were obtained for both medium implants (2 MPa).

The highest decrease of von Mises stress in the micro-screws caused by 30% of osseointegration was observed in S_320_ implant (to 3 MPa), while the lowest variation was for L_320_ (about 4 MPa).

## 4. Discussion

In this study, the influence of bioactive HA coating deposition on Ti6Al4V implant’s biomechanical performance was investigated. As opposed to the non-coated implant held by the fixation mini-plates only, it was assumed that such deposition should result into a higher stability of the implant and its fixation due to additional osseointegration. Such hypothesis was confirmed by the FEM models in both used approaches.

### 4.1. Experimental work

Chemically, the Ca/P ratio in the produced coatings increased significantly (2.45) as compared to the initial pure powder feedstock (1.89). Such increase is frequently observed in plasma deposited HA materials and is triggered by a selective evaporation of phosphorus [29]. This is accompanied by formation of new phases with a higher Ca/P ratio such as tetracalcium phosphate (TTCP, Ca/P 2.00), or even formation of phosphorus-free phases such as CaO [45]. Naturally, the mechanical properties of such multiphase coatings may generally differ from those of pure HA coatings.

The microstructure of the HA coatings was very dense and the individual splats could not be easily recognized. In fact, it was significantly denser than majority of the coatings described in the literature [29, 42, 46]. This was a consequence of the performed single splat impact selection of an appropriate parameters and translated into significantly higher values of mechanical properties (such as Young’s modulus).

The Young’s modulus of the HA coating was determined as 71.8 GPa by the nanoindentation method. As with all thermally sprayed coatings, this value is lower than the value indicated for the corresponding crystalline bulk HA (80–110 GPa [46]). This is caused by the internal porosity and network of micro-cracks. However, at the same time, the value was significantly higher than the values presented in the literature (generally below 20 GPa, e.g. 0.50–5.34 GPa in [42]). Partially, this was given by the dense microstructure of used coatings and the relatively low measured values of internal porosity 8.0 ± 1.0% from the SEM image analysis. The open porosity of 4.6% measured using Archimedean weighing method is a rather small value, again indicating a proper melting of the feedstock HA.

### 4.2 Computational modeling

Overall, 140 computational models (see *Appendix B* in S1 Appendix) were created and analyzed to assess feasibility study of two different computational approaches which mimic osseointegration processes at BIC location. Additionally, computational models of non-coated Ti6Al4V implant were examined to provide mutual comparison of as-fixed states (excluding positive influence of HA coating) of both implant variants–Ti6Al4V + HA coating and non-coated Ti6Al4V.

#### 4.2.1 As-fixed (non-osseointegrated) state

As with other invasive surgical procedures, it is critically important that the used implant fits inside the cranial defect seamlessly in order not to degrade its performance [47] or cause unwanted infections at the BIC interface [1]. Further, the used metallic implants are often somewhat rigid and do not match the flexibility of human bones, which may lead to low ability of energy absorbing while loading [48].

The initial, non-osseointegrated state was identical for both subsequent computational approaches. The discussion in this section is therefore considered valid for both approaches. Two HA coating thicknesses were modeled to comprehend this factor. The results showed that there were no significant differences between these two thicknesses in results of the total implant displacements and von Mises stress in the implants or the fixation components. Considering this, usage of thicker coatings (i.e., increased economy of the deposition process) seem unnecessary since the beneficial–if small at this stage—effect of the coating deposition is manifested for the thinner coating type already.

The results of the investigated von Mises stresses in the implant, the HA coating and the fixation components were relatively low, reaching >15% of the yield stress (yield stress values are listed in Table 1).

#### 4.2.2 Influence of implant osseointegration

The resulting displacement and von Mises stress could be alleviated by a deposition of bioactive coatings onto the metallic implants, providing a secondary fixation and a smoother transition between the mechanical properties of the rigid implant and the surrounding bone. In fact, such phenomenon was already suggested in another study [7]. In the study, the authors have shown that a successful bonding between a cranial bone and an implant can be achieved at BIC. Here, the bone formation was stimulated by material bioactivity. Therefore, the influence of HA coating deposition onto the titanium implants was investigated in this study to assess a possible risk reduction of an implanted components failure.

In the osseointegration process, the newly formed bone initially fills the inevitable gaps between the cranium and the implant. The bone then ingrowths into the provided coating structure, strengthening the fixation between the bone and the implant [48].

In order to address the osseointegration and allow broader understanding of the obtained results, two different computational approaches were employed in this study. The first *Approach I* modeled the osseointegration by a gradual increase in friction at the BIC interface. Upon reaching a pre-selected value of *FC* = 0.75, the total implant displacement decreased noticeably. This is a consequence of the osseointegration process, whereby the HA coating aids in fixing the implant in its position. A similar phenomenon was observed for the von Mises stresses of the implants, fixation mini-plates and the screws. In some combinations, the stress values dropped to virtually zero (compared to the initial values of ~10^2^ MPa). The reduced values are shown in Figs 5, 6 and 9. This presents a favorable effect of the secondary stability (biological stability formed after the formation of a secondary bone contact) [49, 50].

In the *Approach II*, the osseointegration process was modeled by an incremental bonding of the BIC interface contact region. At the pre-selected level of 30% osseointegration, all monitored properties exhibited a significant decrease, both in terms of implant movement (displacement) or the associated stresses. The reduced values presented in Figs 7–9 suggested a significant improvement due to the osseointegration process.

A direct mutual comparison of the results obtained via the two approaches is complicated. In fact, the connection between the values of *FC* (*Approach I*) and percentage of osseointegration (*Approach II*) is not straightforward–the final state of *Approach I* (fully bonded contact at BIC) differed to the rough contact (final state) in *Approach II* by more than 15%. Therefore, it is difficult to assign those to identical levels of osseointegration. Despite the inability of exact mutual comparison between the two approaches, both predict a significant improvement of the displacement and stress situation in the cranioplasty procedure modeled herein. The results were in a good agreement with assumptions—positive impact of osseointegration process amplified by HA coating at BIC.

#### 4.2.3 Benefits of HA coating deposition

To present the benefits of HA coating onto cranial implants, computations of non-coated implant manufactured from Ti6Al4V (small-size implant configuration) were complemented to provide mutual comparison.

The results of non-coated Ti6Al4V implant correspond to the non-osseointegrated state of the coated variants. The beneficial effect of HA coating deposition onto Ti6Al4V substrates could not be recognized immediately after the surgery as the investigated parameters either improved by only a few percent or remained identical. Importantly, this adjustment did not cause any negative effect and upon osseointegration, the monitored parameters further decreased dramatically, presenting the major benefit of the coating process (Table 2).

**Table 2:** A comparison of results obtained for a small-sized cranial Ti6Al4V implant without and with HA coating.

		Ti6Al4V	Ti6Al4V + HA	App. I ^1^	App. II ^2^
**Displacement of implant**	[mm]	0.050	0.050	0.012	0.002
**Stress in implant**	[MPa]	52	50	10	1
**Stress in mini-plates**	[MPa]	102	102	1	0
**Stress in micro-screw**	[MPa]	120	117	1	0
**Implant D&M process**	[time]	*t*_*1*_	*t*_*1*_ + *t*_*2*_	*t*_*1*_ + *t*_*2*_+ *t*_*3*_	*t*_*1*_ + *t*_*2*_+ *t*_*3*_

Both columns represent the as-fixed, non-integrated states, while the columns *App*. *I* and *App*. *II* pertain to fully osseointegrated states of the HA-coated implant computed by the two approaches. The complexity of the process is symbolically shown in total amount of production time, where *t*_*1*_, *t*_*2*_, *t*_*3*_ correspond to design and manufacturing process of the implant, coating deposition, and osseointegration process, respectively (*t*_*3*_ > *t*_*1*_ > *t*_*2*_).

It is important to understand the obtained results against the fact that the spray process presents an additional step in the manufacturing process, bringing along associated increase in the total cost and production time (Table 2). A thorough consideration of all aspects is therefore necessary.

## 5. Conclusion

Nowadays, the production of a patient-specific Ti6Al4V implant coated with HA is enabled by using a combination of 3D printing and plasma spray technologies. This study investigated the benefits of such HA coating amplified by osseointegration on biomechanical properties of a cranial implant.

Two parts were presented, experimental and computational modeling. The former consisted of optimization and plasma spraying of HA coatings and measurement of their properties. This data was then used as an input to FEM modeling, realized as two different computational approaches to comprehend the effect of gradual osseointegration.

The results showed that, directly upon implantation, the HA layer at bone-implant contact area caused only a slight improvement in some monitored parameters (a decrease in the implant displacements and von Mises stresses in the fixation components). However, upon osseointegration, this positive effect intensified significantly: the results of both approaches proved a major reduction of investigated parameters such as the total implant displacements and von Mises stresses in the implanted components (in comparison to non-coated, non-osseointegrated variant). This is a very promising result for potential use of thermally sprayed HA coatings for cranial implants.

## Supporting information

S1 Appendix (DOCX)

S1 Graphical abstract (TIF)

## Abbreviations

AM: additive manufacturing
B: bone
BIC: bone-implant contact
CT: computed tomography
FC: frictional coefficient
FEM: finite element method
HA: hydroxyapatite
HAC: hydroxyapatite coating
I: implant
ICP: intracranial pressure
OSS: osseointegrated
PEEK: polyether ether ketone
PMMA: poly-methyl methacrylate
PS: patient specific
STL: standard tessellation language
