# Supplementary material for: Beneficial osseointegration effect of hydroxyapatite coating on cranial implant – FEM investigation
Source: PLoS One. 2021 Jul 19;16(7):e0254837. doi: 10.1371/journal.pone.0254837 (PMC8289038; doi:10.1371/journal.pone.0254837)
Supplement: S1 Appendix — (DOCX) [file pone.0254837.s001.docx]

Appendix A

The loading and unloading curves from the HA Young’s modulus measurements.


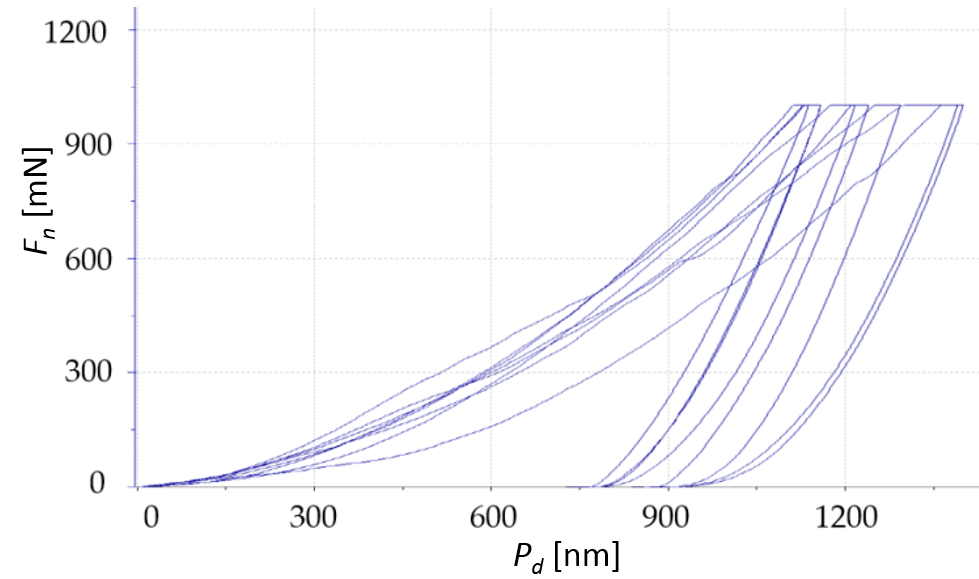


Appendix B

Overall, 140 computational models were created and analyzed (115 coarse models and 25 sub-models – see Table 3).

**Table 3**. The list of all created computational models (coarse models and sub-models).

|  | **Coarse model** | | | **Sub-model** | | |
| --- | --- | --- | --- | --- | --- | --- |
|  | Designation | Qty. | Subtotal | Designation | Qty. | Subtotal |
| *Approach I* | S_160_ | 10 | 60 | S_320_ | 3 | 12 |
|  | S_320_ | 10 |  |  |  |  |
|  | M_160_ | 10 |  | M_160_ | 3 |  |
|  | M_320_ | 10 |  | M_320_ | 3 |  |
|  | L_160_ | 10 |  | L_320_ | 3 |  |
|  | L_320_ | 10 |  |  |  |  |
| *Approach II* | S_160_ | 9 | 54 | S_320_ | 3 | 12 |
|  | S_320_ | 9 |  |  |  |  |
|  | M_160_ | 9 |  | M_160_ | 3 |  |
|  | M_320_ | 9 |  | M_320_ | 3 |  |
|  | L_160_ | 9 |  | L_320_ | 3 |  |
|  | L_320_ | 9 |  |  |  |  |
| *Non-coated Ti6Al4V* | | 1 | 1 | *N-c Ti6Al4V* | 1 | 1 |
| SUM | | 115 | | SUM | 25 | |
