# Supplementary figures and images for: Beneficial osseointegration effect of hydroxyapatite coating on cranial implant – FEM investigation
Source: PLoS One. 2021 Jul 19;16(7):e0254837. doi: 10.1371/journal.pone.0254837 (PMC8289038; doi:10.1371/journal.pone.0254837)

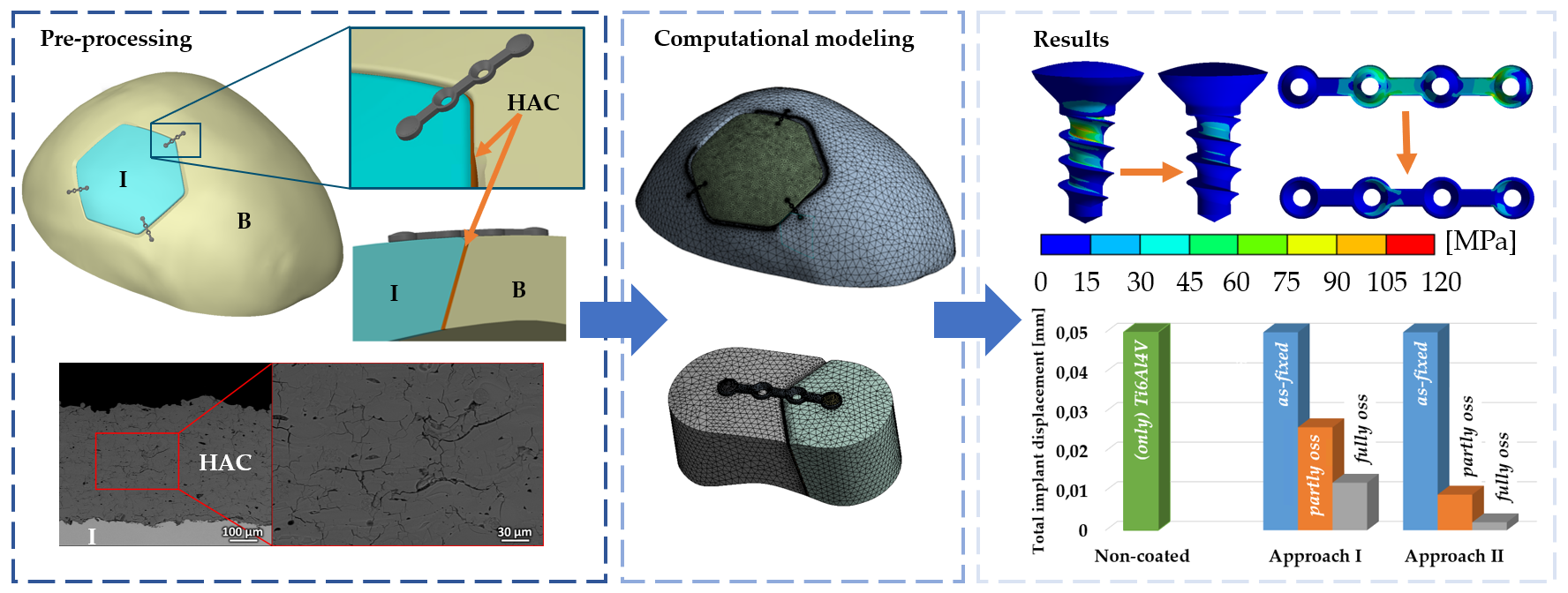

Supplement: S1 Graphical abstract — (TIF) [file pone.0254837.s002.tif]
